# Association between Thyroid Hormone Levels and Diabetic Kidney Disease in Euthyroid Patients with Type 2 Diabetes

**DOI:** 10.1038/s41598-018-22904-7

**Published:** 2018-03-16

**Authors:** Jian Zou, Feng Tian, Yi Zhang, Zeping Li, Chao Yang, Haixu Chen, Jiajia Zhai, Min Shi, Chao Xu, Juan Zhang, Wenjuan Li, Yonghong Xie, Xiaomiao Li

**Affiliations:** 1 Department of Medicine, The 522 Hospital of the Chinese PLA, Luoyang, Henan Province China; 2 Department of Endocrinology and Metabolism, The First Affiliated Hospital of Fourth Military Medical University, Xi’an, Shaanxi Province China; 3 Department of Endocrinology and Metabolism, Xi’an Ninth People’s Hospital, Xi’an, Shaanxi Province China; 4 Queen Marry College, Nanchang University, Nanchang, Jiangxi Province China; 5 Department of Blood Transfusion, General Hospital of the PLA Rocket Force, Beijing, China; 6 Institute of Geriatrics, General Hospital of the Chinese PLA, Beijing, China; 7 Department of Endocrinology and Metabolism, 3201 Hospital, Hanzhong, Shaanxi Province China; 8 Department of Respiratory, The Second Affiliated Hospital of Fourth Military Medical University, Xi’an, Shaanxi Province China

## Abstract

The association between normal thyroid function and diabetic kidney disease (DKD) has gained increasing attention. The present study evaluated the relationship between normal thyroid hormone levels and DKD in type 2 diabetes mellitus (T2DM) patients. A total of 862 type 2 diabetes patients were enrolled in this cross-sectional study in Xi^’^an, Shaanxi Province, China. The subjects were evaluated for anthropometric measurements, thyroid function and DKD. Of 862 patients, 246 (28.5%) suffered from DKD, and the prevalence of DKD did not differ between men and women. The prevalence of DKD showed a significantly decreasing trend across the quartiles based on free triiodothyronine (FT3) levels (41.1%, 30.6%, 23.8%, and 18.9%, P < 0.001). In comparison with all participants categorized in the first FT3 quartile group (FT3-Q1) (<4.380), the adjusted odds ratio of DKD in the second FT3 quartile group (FT3-Q2), the third FT3 quartile group (FT3-Q3), and the fourth FT3 quartile group (FT3-Q4) were 0.655(95%CI: 0.406–1.057), 0.493(95%CI: 0.299–0.813), 0.406(0.237–0.697) (P < 0.05). Also, similar results were observed in men. Conversely, none of the FT3 groups was associated with DKD in women. The present study showed that FT3 within normal range was negatively correlated with DKD in T2DM patients.

## Introduction

The prevalence of diabetes is rising rapidly worldwide, especially in developing countries. Diabetes mellitus is being one of the greatest health threats in the 21^st^ century. Diabetic kidney disease (DKD) is one of the most common microvascular complications of diabetes mellitus and the leading cause of end-stage renal disease (ESRD) worldwide. About 20%–40% of patients with diabetes progress to DKD, and 40% also progress to ESRD^1^. Diabetic patients have a high prevalence of thyroid dysfunction as compared to healthy population; hypothyroidism is the most common disorder^2^. Several clinical studies showed that thyroid dysfunction was related to renal disease^3,4^. In addition, previous studies focused on the relationship between subclinical hypothyroidism (SCH) and DKD^4–6^, although the results were not consistent.

Rodacki *et al*.^7^ found that not only SCH increased the prevalence of DKD but also the high normal levels of thyroid stimulating hormone (TSH) increased the prevalence of DKD as compared to low normal levels in type 1 diabetes (T1DM) patients. Moreover, studies in patients with euthyroid general population showed that high normal levels of TSH and low levels of free triiodothyronine (FT3) were associated with chronic kidney disease (CKD) and albuminuria^8–11^. Therefore, these results indicated that the relationship between thyroid function and DKD had been extended to the areas of normal thyroid function. However, it is unclear whether the normal thyroid hormone levels and DKD are correlated in type 2 diabetes (T2DM) patients.

The present cross-sectional study investigated whether serum thyroid hormone concentrations within normal range, as well as thyroid peroxidase antibody (TPO-Ab) levels, are related to the prevalence of DKD among T2DM inpatients.

## Methods

### Subjects

The study was conducted at the First Affiliated Hospital of Fourth Military Medical University in Xi^’^an, Shaanxi Province, China from June 2014 to March 2016. The inclusion criteria were as follows: individuals with previously diagnosed T2DM and all patients were ≥18 years of age. The American Diabetes Association (ADA) criteria 2013^12^ were used for the diagnosis of diabetes mellitus. T2DM was defined as an fasting plasma glucose (FPG) level ≥126 mg/dL (7.0 mmol/L), or oral glucose tolerance test ≥200 mg/dL (11.1 mmol/L), or HbA1c ≥48 mmol/mol (6.5%), or a history of T2DM based on the ADA 2013 criteria^12^. Duration of diabetes is defined in this presentation as time of diagnosis to entry into the trial^13^.

The following exclusion criteria were considered: (1) other types of diabetes mellitus; (2) acute intercurrent illness; (3) severe cardiac disease and other serious diseases; (4) urinary tract infections, hematuria (including menstrual period), and non-diabetic kidney disease; (5) chronic diseases that can affect the metabolic function, including hypothalamic disease, adrenal disease, history of thyroid disease, or any thyroid medication (levothyroxine or anti-thyroid drugs); (6) malignant tumors. Initially, 2689 inpatients were enrolled. After excluding the subjects that did not fulfill the inclusion criteria and those with incomplete laboratory results, a total of 862 euthyroid patients were included in the analysis. The study protocol was approved by the Ethics Committee of the First Affiliated Hospital of Fourth Military Medical University and conducted in accordance with the Declaration of Helsinki. All participants provided written informed consent.

### Data collection

Patient data, including demographic characteristics, lifestyle habits (smoking and drinking), medical history, medication of diabetes, duration of diabetes, thyroid disease, and all results of tests and examinations were obtained from the discharged medical records. Body mass index (BMI) was calculated by the formula: body weight (kg)/height (m), and waist-hip ratio (WHtR) was calculated by waist (cm)/hip (cm). The blood pressure was measured with a cuff in the sitting position after a rest period of more than 10 min. Hypertension was defined by a systolic blood pressure (SBP) ≥140 mmHg or a diastolic blood pressure (DBP) ≥90 mmHg or both, or if the patient had already administered anti-hypertensive drugs. The subjects were requested to fast for a minimum of 8–12 h and avoid a high-fat diet or alcohol consumption for at least 24 h.

Also, the results were obtained for the following variables: FPG, serum creatinine (SCr), total serum cholesterol (TC), serum triglyceride (TG), serum high-density lipoprotein cholesterol (HDL-C), serum low-density lipoprotein cholesterol (LDL-C), and HbA1c. The thyroid function was confirmed by chemiluminescence immunoassay (ADVIA Centaur Siemens New York, USA). The measuring reference ranges of serum TSH, FT3, free thyroxine (FT4), and TPO-Ab were 0.35–5.5 μIU/mL, 3.5–6.5 pmol/L, 11.5–22.7 pmol/L, and 0–78 U/mL, respectively. As described in a previous publication^14^, the subjects were also divided into 4 groups according to the quartiles of FT3 and FT4 in the present study. Meanwhile, the National Academy of Clinical Biochemistry (NACB) recommended lowering the upper reference limit of TSH to 2.5 uIU/mL based on a large-scale epidemiological survey in 2003, which stated that >95% of the normal individuals presented TSH levels <2.5 uIU/mL, and those with high TSH levels were likely to experience various thyroid disorders^15^. In addition, several similar investigations^7,16,17^ divided the participants within the normal range of TSH into TSH <2.5μIU/mL and TSH ≥2.5 μIU/mL groups. Thus, we divided the subjects into 2 groups according to TSH <2.5μIU/mL and TSH ≥2.5 μIU/mL. To our knowledge, there is currently no study on the relationship between TPO-Ab and DKD. Moreover, as described in a previous investigation^18^, the subjects were divided into 2 groups according to negative of TPO-Ab and positive of TPO-Ab. The urine specimens were collected using the first urine sample in the morning. Before the examination, the patients were instructed to avoid exercise for 1 h. The urinary albumin-to-creatinine ratio (UACR), <3 mg/mmol is defined as normal^19^.

### Assessment of eGFR, SCH, and DKD

Estimated glomerular filtration rate (eGFR) was calculated using a modified MDRD equation in the Chinese population^20^ as follows: ${\rm{eGFR}}=175\times {{SCr}}^{-1.234}\times {{age}}^{-0.179}(\times 0.79{females})$. SCH was defined as normal FT4 and FT3 levels and an elevated TSH level^21^. According to the 2012 Kidney Disease Outcomes Quality Initiative (KDOQI) guidelines^22^ and Diabetic Kidney Disease Consensus in China-2014^19^, DKD referred to CKD caused by diabetes that was defined as glomerular filtration rate(GFR) <60 mL/(min × 1.73 m^2^) or UACR >3 mg/mmol for more than 3 months, excluding non-DKD.

### Statistical analysis

Statistical analyses were performed using SPSS 16.0 statistics software (SPSS Inc., Chicago, IL, USA). Continuous variables were expressed as mean ± standard deviation or median (interquartile range), and categorical variables were expressed as percentages. Continuous data were compared using Student’s *t*-test or Mann–Whitney U test, and categorical data were compared by chi-square test. The multiple logistic regression models were used to examine the relationships between TSH, FT3, FT4, and TPO-Ab and the prevalence of DKD with adjustment. Lots of similar investigations^16,23–26^ selected age, gender, BMI, smoking status, duration of T2DM, HbA1c,hypertension, TC, TG and angiotensin Receptor Blocker (ARB) or angiotensin converting enzyme inhibitors (ACEI) medication as the covariates. These studies^16,23–27^ showed that the above covariates do have a substantial impact on the dependent variable. In the present study, compared to the non-DKD patients, those with DKD had higher insulin medication rate (P < 0.05). In addition, metformin is known to cause a decrease in the TSH levels in T2DM patients^28,29^. Thus, the above covariates were adjusted in the logistic regression analysis. Odds ratios (ORs) with their corresponding 95% CI were calculated. All statistical assessments were two-sided, and a P-value < 0.05 was considered statistically significant.

## Results

### Characteristics of the study population

The general characteristics are shown in Tables 1 and 2. A total of 862 subjects were included in this study and 73.8% of the participants were men (n = 636). The mean age was 53.70 ± 11.56 years, and BMI was 25.92 ± 3.35 kg/m^2^. Among 862 patients, 246 (28.5%) were diagnosed with DKD. No significant differences were observed between non-DKD and DKD groups with respect to gender, BMI, TC, LDL-C, and HDL-C. Compared to the non-DKD patients, those with DKD were older with a prolonged duration of diabetes, higher prevalence of hypertension, higher ARB or ACEI medication, higher use of insulin, higher WHtR, SBP, DBP, HbA1c, FPG, TG and lower Hemoglobin (HGB) (all P < 0.05). Moreover, the differences in smoking and drinking status were significantly different among the groups (P = 0.002, P < 0.001, respectively). Subjects with DKD presented a significantly high UACR, uric acid (UA), SCr, and Cystatin C (Cysc) and low eGFR and FT3 (P < 0.05). However, the TSH levels (P = 0.386), FT4 levels (P = 0.302), and the rate of TPO-Ab positive (P = 0.781) were not significantly different between the DKD and non-DKD groups.

**Table 1:** Comparison of clinical characteristics between the non-DKD and DKD groups

Characteristics	Total (n = 862)	Non-DKD(n = 616)	DKD (n = 246)	P value
Age (years)	53.70 ± 11.56	51.98 ± 11.10	58.00 ± 11.59	<0.001
Gender (men %)	636 (73.8)	462 (75.0)	174 (70.7)	0.200
Duration of T2DM (years)	8.75 ± 6.61	7.73 ± 6.26	11.31 ± 6.77	0.001
Smoking n (%)				0.002
No smoking	441 (51.2)	315 (51.1)	126 (51.2)	
Smoking	334 (38.7)	252 (40.9)	82 (33.3)	
Quit smoking	87 (10.1)	49 (8.0)	38 (15.5)	
Drinking n, (%)				<0.001
No drinking	601 (69.7)	407 (66.1)	194 (78.9)	
Drinking	225 (26.1)	187 (30.3)	38 (15.4)	
Quit drinking	36 (4.2)	22 (3.6)	14 (5.7)	
Hypertension, n (%)	381 (44.2)	212 (34.4)	169 (68.7)	<0.001
ARB/ACEI, n (%)	189 (21.9)	100 (16.2)	89 (36.2)	<0.001
Insulin, n (%)	497 (57.7)	322 (52.3)	175 (71.1)	0.001
Metformin, n (%)	487 (56.5)	371 (60.2)	116 (47.2)	0.001
BMI (Kg/m^2^)	25.92 ± 3.35	25.83 ± 3.20	26.15 ± 3.70	0.243
WHtR	0.93 ± 0.06	0.92 ± 0.06	0.93 ± 0.06	0.016
SBP(mmHg)	129.76 ± 17.26	126.01 ± 15.67	139.16 ± 17.51	<0.001
DBP(mmHg)	80.00 ± 10.80	78.98 ± 10.42	82.57 ± 11.32	<0.001
HbA1c (%)	8.61 ± 1.85	8.51 ± 1.85	8.87 ± 1.84	0.009
FPG (mmol/l)	8.54 ± 2.83	8.28 ± 2.76	9.19 ± 2.90	<0.001
TC (mmol/l)	4.04 ± 0.93	4.02 ± 0.89	4.11 ± 1.03	0.226
TG (mmol/l)	1.47(1.02,2.30)	1.39 (1.01,2.25)	1.68 (1.10, 2.44)	0.009
LDL-C (mmol/l)	2.35 ± 0.75	2.34 ± 0.72	2.36 ± 0.80	0.807
HDL-C (mmol/l)	0.97 ± 0.25	0.97 ± 0.23	0.97 ± 0.28	0.814
HGB (g/L)	141.26 ± 15.45	143.39 ± 14.67	135.92 ± 16.06	<0.001
UA (mmol/l)	276.33 ± 78.03	270.14 ± 73.89	291.84 ± 85.75	0.001
SCr (mg/dl)	1.05 ± 0.23	1.00 ± 0.13	1.18 ± 0.33	<0.001
Cysc (mg/dl)	0.90 ± 0.23	0.83 ± 0.13	1.05 ± 0.34	<0.001
eGFR (ml/min.1.73 m^2^)	79.41 ± 16.22	83.32 ± 12.68	69.92 ± 19.64	<0.001
TSH (μIU/ml)	1.94(1.32,2.75)	1.95 (1.31, 2.70)	1.93 (1.33,2.92)	0.386
FT3 (pmol/L)	4.74 ± 0.52	4.80 ± 0.50	4.60 ± 0.55	<0.001
FT4 (pmol/L)	16.34 ± 2.10	16.39 ± 2.11	16.22 ± 2.09	0.302
TPO-Ab, n (%)	69(8.0)	48 (7.8)	21 (8.5)	0.781
UACR (mg/mmol)	1.08(0.63,2.99)	0.82 (0.54, 1.28)	9.36 (3.95, 33.00)	<0.001

Values are expressed as means ± SD or median (range) or count and percentage. ACEI, angiotensin-converting enzyme inhibitor; ARB, angiotensin II receptor blocker; BMI, body mass index; WHtR, waist-to-height ratio; SBP, systolic blood pressure; DBP, diastolic blood pressure; FPG, fasting plasma glucose; TC, total cholesterol; TG, triglycerides; LDL-C, low-density lipoprotein cholesterol; HDL-C, high-density lipoprotein cholesterol; HGB, hemoglobin; UA, uric acid; SCr, serum creatinine; eGFR, estimated glomerular filtration rate; UACR, urinary albumin-creatinine ratio; DKD, diabetic kidney disease. The P-value was obtained by chi-square or Mann–Whitney U test or independent two-sample t-tests.

**Table 2:** Comparison of clinical characteristics between men and women groups.

Characteristics	Total (n = 862)	Men (n = 636)	Women (n = 226)	P value
Age (years)	53.70 ± 11.56	52.20 ± 11.21	57.92 ± 11.51	<0.001
Duration of T2DM (years)	8.75 ± 6.61	8.26 ± 6.39	10.13 ± 7.03	0.001
Smoking, n (%)				<0.001
No smoking	441 (51.2)	226 (35.5)	215 (95.1)	
Smoking	334 (38.7)	323 (50.8)	11 (4.9)	
Quit smoking	87 (10.1)	87 (13.7)	0 (0)	
Drinking, n (%)				<0.001
No drinking	601 (69.7)	379 (59.6)	222 (98.2)	
Drinking	225 (26.1)	222 (34.9)	3 (1.3)	
Quit drinking	36 (4.2)	35 (5.5)	1 (0.5)	
Hypertension, n (%)	381(44.2)	272(42.8)	109(48.2)	0.161
ARB/ACEI, n (%)	189(21.9)	132(20.8)	57(25.2)	0.190
Insulin, n (%)	497(57.7)	345(54.2)	152(67.3)	0.001
Metformin, n (%)	487(56.5)	365(57.4)	122(54.0)	0.391
BMI (Kg/m^2^)	25.92 ± 3.35	26.08 ± 3.08	25.48 ± 4.00	0.043
WHtR	0.93 ± 0.06	0.93 ± 0.06	0.92 ± 0.06	0.001
SBP (mmHg)	129.76 ± 17.26	129.08 ± 16.84	131.68 ± 18.29	0.063
DBP (mmHg)	80.00 ± 10.80	80.71 ± 11.04	78.00 ± 9.87	0.001
HbA1c (%)	8.61 ± 1.85	8.61 ± 1.80	8.60 ± 1.99	0.942
FPG (mmol/l)	8.54 ± 2.83	8.55 ± 2.81	8.50 ± 2.89	0.813
TC (mmol/l)	4.04 ± 0.93	3.98 ± 0.93	4.21 ± 0.91	0.002
TG (mmol/l)	1.47(1.02,2.30)	1.49(1.01,2.36)	1.40(1.03,2.18)	0.198
LDL-C (mmol/l)	2.35 ± 0.75	2.33 ± 0.74	2.39 ± 0.76	0.363
HDL-C (mmol/l)	0.97 ± 0.25	0.92 ± 0.21	1.11 ± 0.28	<0.001
HGB (g/L)	141.26 ± 15.45	145.38 ± 14.21	129.65 ± 12.63	<0.001
UA (mmol/l)	276.33 ± 78.03	289.40 ± 76.44	239.56 ± 70.50	<0.001
SCr (mg/dl)	1.05 ± 0.23	1.09 ± 0.23	0.94 ± 0.18	<0.001
Cysc (mg/dl)	0.90 ± 0.23	0.90 ± 0.22	0.89 ± 0.25	0.536
eGFR (ml/min.m2)	79.41 ± 16.22	80.86 ± 16.08	75.34 ± 15.94	<0.001
TSH (uIU/ml)	1.94(1.32,2.75)	1.86(1.29,2.60)	2.32(1.44,3.17)	<0.001
FT3 (pmol/L)	4.74 ± 0.52	4.83 ± 0.50	4.50 ± 0.50	<0.001
FT4 (pmol/L)	16.34 ± 2.10	16.38 ± 2.10	16.23 ± 2.10	0.374
TPO-Ab, n (%)	69(8.0%)	36(5.7%)	33(14.6%)	<0.001
DKD, n (%)	246(28.5%)	174(27.4%)	72(31.9%)	0.200
UACR (mg/mmol)	1.08(0.63,2.99)	1.04(0.61,3.07)	1.21(0.75,2.86)	0.029

Values are expressed as means ± SD or median (range) or count and percentage. ACEI, angiotensin-converting enzyme inhibitor; ARB, angiotensin II receptor blocker; BMI, body mass index; WHtR, waist-to-height ratio; SBP, systolic blood pressure; DBP, diastolic blood pressure; FPG, fasting plasma glucose; TC, total cholesterol; TG, triglycerides; LDL-C, low-density lipoprotein cholesterol; HDL-C, high-density lipoprotein cholesterol; HGB, hemoglobin; UA, uric acid; SCr, serum creatinine; eGFR, estimated glomerular filtration rate; UACR, urinary albumin-creatinine ratio; DKD, diabetic kidney disease. The P-value was obtained by chi-square or Mann–Whitney U test or independent two-sample t-tests.

The prevalence of DKD did not differ between men and women (27.4% versus 31.9%, P > 0.05). Men were prone to excessive smoking and drinking, higher BMI, WHtR, DBP, HGB, UA, SCr, eGFR, and UACR, lower age, duration of T2DM, TC, and HDL-C (P < 0.05). Moreover, men used insulin to a lower extent than women (P = 0.001). Furthermore, men yielded significantly lower TSH levels and positive rate of TPO-Ab than women (P < 0.001). The former also showed higher FT3 levels than the latter (P < 0.001). However, the rest of the parameters were not statistically significant (Table 2).

### Association of thyroid hormone levels and TPO-Ab with the prevalence of DKD

**Figure 1:**
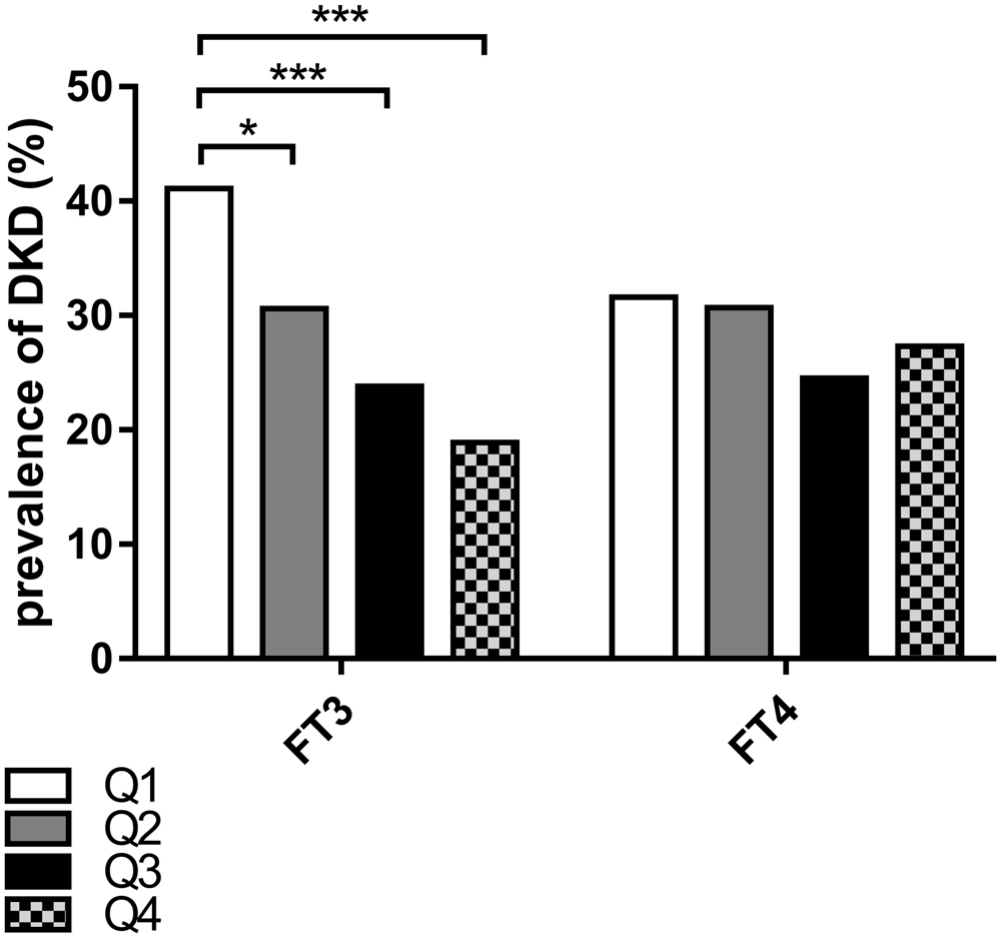
Prevalence of DKD among quartiles based on FT3 and FT4 (*p < 0.05, ***p < 0.001) Q1: the first FT3 (FT4) quartile group, Q2: the second FT3 (FT4) quartile group, Q3: the third FT3 (FT4) quartile group, Q4: the fourth FT3 (FT4) quartile group.

**Figure 2:**
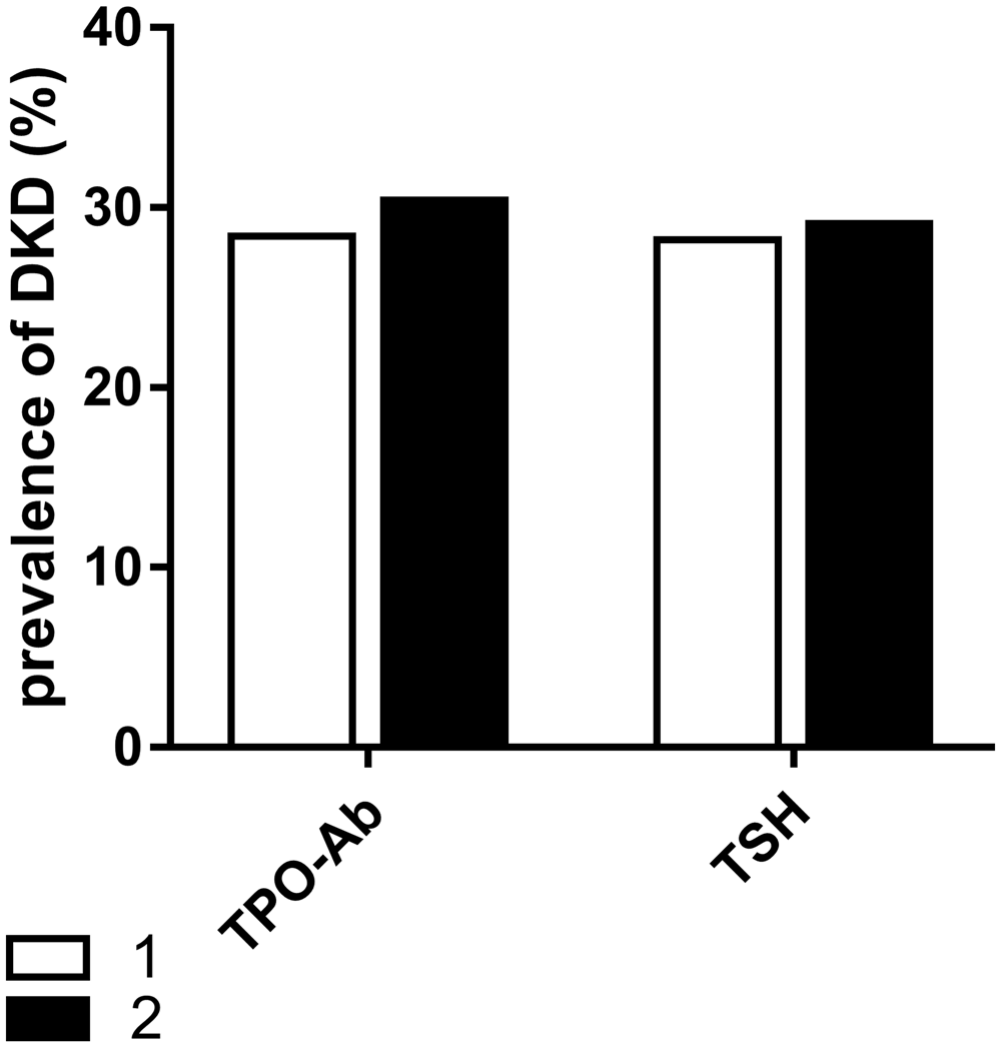
Prevalence of DKD among groups based on TSH levels and TPO-Ab. TSH1: <2.5 μIU/mL, TSH2 ≥2.5 μIU/mL, TPO-Ab1 < 78 U/mL, TPO-Ab2 ≥ 78 U/ml).

In order to explore the association of thyroid function with DKD, we divided the patients into four groups according to the quartiles of FT3 (<4.38, 4.38–4.73, 4.73–5.12 and ≥5.12pmol/L) and FT4 (<14.84, 14.84–16.13, 16.13–17.66 and ≥17.66 pmol/L) and divided two groups based on TSH (<2.5 and ≥2.50 mIU/L) and TPO-Ab levels (<78 U/mL, ≥78U/ml), respectively. The prevalence of DKD showed a significantly decreasing trend across the quartiles based on the FT3 levels (41.1%, 30.6%, 23.8 and 18.9%, p < 0.001 for the trend). The first FT3 quartile group (FT3-Q1) showed a significantly higher prevalence of DKD than the second FT3 quartile group (FT3-Q2), the third FT3 quartile group (FT3-Q3), and the fourth FT3 quartile group (FT3-Q4) (P = 0.027, P < 0.001 and P < 0.001; Fig. 1) (Fig. 1). The prevalence of DKD did not differ significantly among FT4, TSH, and TPO-Ab groups (Figs 1 and 2).

### Relationship of thyroid hormone levels and TPO-Ab with the prevalence of DKD

The crude and adjusted relationships between FT3, FT4, TSH, TPO-Ab, and the prevalence of DKD in total population, women, and men are indicated in Tables 3, 4, and 5, respectively. In comparison to all participants categorized in FT3-Q1 (<4.380) (Table 2), the crude ORs for DKD of all participants in FT3-Q2, FT3-Q3, and FT3-Q4 (4.380–4.730, 4.730–5.120, ≥5.120)were 0.632 (95%CI: 0.425–0.939), 0.447 (95%CI: 0.295–0.680), 0.333 (95%CI: 0.215–0.516) (P = 0.023, P < 0.001, P < 0.001) respectively. After an adjustment of the potential factors, including age, gender, duration of T2DM, BMI, smoking, hypertension, ARB/ACEI medication, insulin medication, metformin medication, HbA1c, TG, and TC, the ORs for DKD in FT3-Q2, FT3-Q3, and FT3-Q4 were 0.605 (95%CI: (0.382–0.957), 0.516 (95%CI: 0.317–0.838), 0.471 (95%CI: 0.280–0.792) (P = 0.032, P = 0.008, P = 0.005). However, none of the ORs were statistically significant with respect to the TSH, FT4, and TPO-Ab levels.

**Table 3:** Relationship between thyroid hormone levels and DKD in total population.

Characteristics	Groups	n	Model1 OR (95%CI)	P value	Model2 OR (95%CI)	P value
TSH	<2.5	577	Reference		Reference	
	≥2.50	285	1.044(0.763–1.428)	0.789	0.804(0.551–1.173)	0.258
FT3	Q1(<4.38)	209	Reference		Reference	
	Q2(4.38 ≤ Q2<4.73)	222	0.632(0.425–0.939)	0.023	0.605(0.382–0.957)	0.032
	Q3(4.73 ≤ Q3<5.12)	214	0.447(0.295–0.680)	<0.001	0.516(0.317–0.838)	0.008
	Q4(≥5.12)	217	0.333(0.215–0.516)	<0.001	0.471(0.280–0.792)	0.005
FT4	Q1(<14.84)	215	Reference		Reference	
	Q2(14.84 ≤ Q2<16.13)	215	0.958(0.637–1.440)	0.835	1.005(0.627–1.610)	0.984
	Q3(16.13 ≤ Q3<17.66)	212	0.703(0.459–1.075)	0.103	0.668(0.410–1.089)	0.105
	Q4(≥17.66)	220	0.811(0.536–1.225)	0.319	1.066(0.657–1.729)	0.797
TPO	<78	793	Reference		Reference	
	≥78	69	1.104(0.646–1.887)	0.716	0.982(0.523–1.846)	0.956

Model1: unadjusted Model2: adjusted for age, gender, duration of T2DM, BMI, smoking, hypertension, ARB/ACEI medication, insulin medication, metformin medication, HbA1c, TG, TC.

**Table 4:** Relationship between thyroid hormone levels and DKD in women.

Characteristics	Groups	n	Model1 OR (95%CI)	P value	Model2 OR (95%CI)	P value
TSH	<2.5	125	Reference		Reference	
	≥2.50	101	1.369(0.781–2.402)	0.273	1.251(0.627–2.495)	0.524
FT3	Q1(<4.15)	54	Reference		Reference	
	Q2(4.15 ≤ Q2<4.48)	58	0.518(0.238–1.130)	0.098	0.506(0.191–1.341)	0.171
	Q3(4.48 ≤ Q3<4.80)	56	0.500(0.227–1.102)	0.086	0.483(0.182–1.283)	0.144
	Q4(≥4.80)	58	0.436(0.197–0.967)	0.041	0.413(0.152–1.119)	0.082
FT4	Q1(<14.56)	56	Reference		Reference	
	Q2(14.56 ≤ Q2<16.06)	57	1.056(0.481–2.316)	0.893	0.912(0.319–2.608)	0.864
	Q3(16.06 ≤ Q3<17.54)	57	0.824(0.368–1.843)	0.637	0.582(0.204–1.664)	0.313
	Q4(≥17.54)	56	1.084(0.493–2.383)	0.841	1.781(0.657–4.828)	0.256
TPO	<78	193	Reference		Reference	
	≥78	33	0.919(0.412–2.048)	0.836	1.120(0.408–3.079)	0.825

Model1: unadjusted;

Model2: adjusted for age, duration of T2DM, BMI, smoking, Hypertension, ARB/ACEI medication, insulin medication, metformin medication, HbA1c, TG, TC.

**Table 5:** Relationship between thyroid hormone levels and DKD in men

Characteristics	Groups	n	Model1 OR (95%CI)	P value	Model2 OR (95%CI)	P value
TSH	<2.5	412	Reference		Reference	
	≥2.50	224	1.035(0.720–1.488)	0.851	0.863(0.565–1.317)	0.495
FT3	Q1(<4.38)	156	Reference		Reference	
	Q2(4.38 ≤ Q2<4.69)	175	0.577(0.362–0.920)	0.021	0.569(0.333–0.973)	0.040
	Q3(4.69 ≤ Q3<5.10)	153	0.396(0.243–0.645)	<0.001	0.471(0.271–0.819)	0.008
	Q4(≥5.10)	152	0.273(0.162–0.459)	<0.001	0.384(0.209–0.708)	0.002
FT4	Q1(<14.99)	145	Reference		Reference	
	Q2(14.99 ≤ Q2<16.17)	167	1.033(0.640–1.668)	0.894	1.026(0.591–1.781)	0.927
	Q3(16.17 ≤ Q3<17.66)	160	0.734(0.444–1.214)	0.228	0.681(0.381–1.216)	0.194
	Q4(≥17.66)	164	0.832(0.511–1.354)	0.459	1.110(0.626–1.970)	0.720
TPO	<78	590	Reference		Reference	
	≥78	46	1.433(0.761–2.700)	0.265	1.350(0.636–2.866)	0.434

Model1: unadjusted;

Model2: adjusted for age, duration of T2DM, BMI, smoking, Hypertension, ARB/ACEI medication, insulin medication, metformin medication, HbA1c, TG, TC.

In women, Table 4 showed that in the unadjusted model, compared to the FT3-Q1 (<4.15) (Table 3), the crude OR for DKD in FT3-Q4 (≥4.80) was 0.436 (0.197–0.967) (P = 0.041). However, after further adjustment of the potentially confounding factors, none of the FT3 quartiles was associated with DKD. In men, compared to the FT3-Q1 (<4.38) (Table 5), the crude ORs for DKD in FT3-Q2, FT3-Q3, and FT3-Q4 (4.38–4.69, 4.69–5.10, ≥ 5.10) were significantly different [0.577(0.362–0.920), 0.396(0.243–0.645), 0.273(0.162–0.459)]. After further adjusting the potential factors, the adjusted ORs for DKD were significant [0.569(0.333–0.973), 0.471(0.271–0.819), 0.384(0.209–0.708)]. The crude and adjusted ORs between FT4, TSH, TPO-Ab and DKD had no significant differences in men and in women, respectively.

## Discussion

A total of 862 T2DM patients were enrolled in the cross-sectional study. The prevalence of DKD was 28.5% among T2DM patients, and no significance was observed between men and women. Thus, a higher prevalence of thyroid disease was observed in women as compared to men^30^. Therefore, we grouped men and women to discuss the association between thyroid hormones and DKD, respectively. Moreover, the prevalence of DKD decreased gradually as the quartiles of FT3 levels increased. The results of the current logistic regression analysis also revealed that FT3 levels were inversely associated with the prevalence of DKD in total population and men and not only in unadjusted models but also adjusted models. However, no significance was observed between FT3 and DKD in women. Therefore, FT3 levels exhibited a potentially significant association with DKD in euthyroid patients with type 2 diabetes.

Thyroid hormones participate in the physiological function of the kidney. Thyroid dysfunction can cause changes in renal blood flow, glomerular filtration rate, renal tubular absorption and secretion, and renal structure^31^. Furthermore, several studies showed that thyroid hormone levels within the normal range were associated with the risk of CKD^9–11^. A cohort study also indicated that low normal FT3 levels were associated with an increased risk of CKD in euthyroid function population^9^. Another study in middle-aged and elderly Chinese individuals found that low normal FT3 levels were associated with a high prevalence of microalbuminuria^8^. A small-scale study showed that the total triiodothyronine (TT3) level was significantly correlated with the levels of microalbuminuria. However, whether or not the thyroid hormone levels were within the normal range remained unclear in this study, while the FT3 levels had not been detected^32^. The above studies suggested that low normal FT3 levels were associated with CKD. However, only a few studies focused on the relationship between normal thyroid hormone levels and DKD in T2DM. Hitherto, only one study found a negative correlation between FT3 levels and DKD incidence in T2DM patients with normal thyroid function^33^. Our results were in agreement with the above study: FT3 levels were negatively correlated with the prevalence of DKD in T2DM patients with normal thyroid function. The current study failed to show a significant association between normal levels of FT3 and DKD in the women population. The potential reasons are illustrated as follows. Firstly, sex hormones (such as estrogen and testosterone) can regulate the thyroid function^34^. The gender difference in the relationship between FT3 and DKD can partially be explained by the difference in sex hormones. We did not measure levels of sex hormones in this study, which necessitates further research. Secondly, since the sample size of women was smaller than men, the precision and statistical power of the analysis might be lower for women. Additionally, the proportion of men was higher than that of women, which could also influence the correlation between thyroid hormone levels and DKD in women patients. Lastly, the FT3 levels of the women population were significantly lower than that in men (P < 0.001). A previous study^23^ in China based on the relationship between thyroid hormone and T2DM speculated that the prevalence of DKD depended on the concentrations of FT3 irrespective of the gender. Thus, further large-scale studies are required to clarify this hypothesis.

The present study had several characteristics. Firstly, men were prone to suffering from DKD than women^3^; however, the prevalence of DKD was higher in women than men without any statistical significance in the present study (31.9% vs 27.4%, P = 0.200). The possible explanations were as follows: the age of the women patients in the population was older than men (57.92 ± 11.51, women; 52.20 ± 11.21, men; P < 0.001), and the duration of T2DM of the former was longer than the latter (10.13 ± 7.03, women; 8.26 ± 6.39, men; P = 0.001). In addition, age and duration of diabetes are also risk factors for DKD^3^. Furthermore, the characteristics of the population in this study reflected the current situation of T2DM patients in Northwest China. Secondly, in this study, the “DKD” term was defined according to the guidelines of 2012 KDOQI^22^ and Diabetic Kidney Disease Consensus in China-2014^19^: DKD was diagnosed with either GFR or UACR. Nevertheless, previous studies continue the usage of “dabetic nephropathy (DN)” term, and the value of UACR to diagnose DN, missing having normal UACR value and abnormal eGFR value patients.

The mechanism underlying the association between FT3 and DKD could be clearly demonstrated by the following: Firstly, T3 had been shown to influence the endothelial function by relaxation the vascular smooth muscle cells through direct or indirect effects in experimental models^35–37^. Low T3 was closely relevant to endothelial dysfunction in patients with advanced non-DKD^38^. The study confirmed that serum TSH levels in the upper reference range were also associated with impaired endothelial function measured via flow-mediated dilation^39^. The endothelial dysfunction was associated with albuminuria in diabetes^40^; therefore, low FT3 level and albuminuria may be related to endothelial dysfunction. Secondly, T3 could attenuate albuminuria and improve the renal structural damage in db/db diabetic mice by increasing the activity of phosphatidylinositol 3 kinase and decreasing the expression of transforming growth factor-β1^41^. Finally, 3,5-diiodothyronine could protect the cells from renal damage in DKD by inhibiting the activation of NF-κβ and JNK^42^. These enzymes and pathways, mentioned above, were involved in the development of DKD.

However, the present study failed to find a correlation between TSH and DKD. The current results were in agreement with the study by Wu *et al*.^33^; however, the results differed from those reported by Qi *et al*.^16^ demonstrated an association between high TSH levels and an increased risk of DKD. Currently, the reasons for this discrepancy are yet to be elucidated, which might be related to differences in demographic and clinical characteristics, ethnicity, and research design. These phenomena might be attributed to the following: Patients with positive TPO-Ab may not be excluded in the present study. The population with positive TPO-Ab has a higher TSH level than the negative TPO-Ab in euthyroid population, and the TPO-Ab levels may be responsible for the endothelial dysfunction and subsequent microalbuminuria^43^. On the other hand, metformin is known to cause a decrease in the TSH levels in T2DM patients^28,29^. Thus, metformin is widely used in T2DM patients as a first-line treatment. In the current analysis, we adjusted the rate of metformin usage; however, these factors may influence the association between TSH and DKD. None of the ORs were found to be significant in FT4 quartiles, and this result was consistent with the previous study^33^, which might be attributed to the role of thyroid hormones that is achieved by FT3 binding to receptors and other related proteins to regulate the transcription of target genes and the expression of proteins. Similarly, no relationship between TPO-Ab and DKD was found in this study, which might be associated with a low positive TPO-Ab rate (8.0%). Moreover, previous studies indicated that T1DM with thyroid dysfunction was mostly due to the presence of autoimmune diseases and T2DM with thyroid dysfunction was primarily due to insulin resistance^44,45^.

In conclusion, the present study showed that FT3 in normal range was negatively correlated with DKD in T2DM with normal thyroid function. These results could aid in predicting the risk of DKD development, as well as the basis for a large-scale cohort study.

Nevertheless, the present study has some limitations. Firstly, this was a cross-sectional study with lack of long-term follow-up; thus, the relative risk could not be assessed. Therefore, further prospective and longitudinal studies should be conducted to confirm the relative risk between FT3 levels within normal range and DKD in T2DM patients. Secondly, UACR and thyroid function were estimated only once, which could result in misleading classifications. Thirdly, we did not assess the reverse triiodothyronine (rT3) of the population, and diabetes may have a low triiodothyronine (T3) syndrome, which is prone to hypothyroidism^46^, and thus, the relationship between hypothyroidism and some factors may have been underestimated in this study. Furthermore, the proportion of men was significantly higher than that of women, which might affect the relationship between the levels of thyroid hormone and DKD in women patients.

## References

[1] Molitch ME (2004). Nephropathy in diabetes. Diabetes care.

[2] Wu P (2007). Thyroid disorders and diabetes. It is common for a person to be affected by both thyroid disease and diabetes. Diabetes self-management.

[3] Furukawa S (2014). Association between subclinical hypothyroidism and diabetic nephropathy in patients with type 2 diabetes mellitus. Endocrine journal.

[4] Chen HS (2007). Subclinical hypothyroidism is a risk factor for nephropathy and cardiovascular diseases in Type 2 diabetic patients. Diabetic medicine: a journal of the British Diabetic Association.

[5] Yasuda T (2011). Subclinical hypothyroidism is independently associated with albuminuria in people with type 2 diabetes. Diabetes research and clinical practice.

[6] Kim BY (2011). Association between subclinical hypothyroidism and severe diabetic retinopathy in Korean patients with type 2 diabetes. Endocrine journal.

[7] Rodacki M (2014). Should thyroid-stimulating hormone goals be reviewed in patients with type 1 diabetes mellitus? Results from the Brazilian Type 1 Diabetes Study Group. Diabetic medicine: a journal of the British Diabetic Association.

[8] Zhou Y (2014). Free triiodothyronine concentrations are inversely associated with microalbuminuria. International journal of endocrinology.

[9] Zhang Y (2014). Thyroid hormone levels and incident chronic kidney disease in euthyroid individuals: the Kangbuk Samsung Health Study. International journal of epidemiology.

[10] Sun MT, Hsiao FC, Su SC, Pei D, Hung YJ (2012). Thyrotropin as an independent factor of renal function and chronic kidney disease in normoglycemic euthyroid adults. Endocrine research.

[11] Asvold BO, Bjoro T, Vatten LJ (2011). Association of thyroid function with estimated glomerular filtration rate in a population-based study: the HUNT study. European journal of endocrinology.

[12] American Diabetes, A. (2013). Standards of medical care in diabetes–2013. Diabetes care.

[13] Duckworth WC (2011). The duration of diabetes affects the response to intensive glucose control in type 2 subjects: the VA Diabetes Trial. Journal of diabetes and its complications.

[14] Zhou Y (2016). Free Triiodothyronine Concentrations are Inversely Associated with Elevated Carotid Intima-Media Thickness in Middle-Aged and Elderly Chinese Population. Journal of atherosclerosis and thrombosis.

[15] Kratzsch J (2005). New reference intervals for thyrotropin and thyroid hormones based on National Academy of Clinical Biochemistry criteria and regular ultrasonography of the thyroid. Clinical chemistry.

[16] Qi Q (2017). Association of Thyroid-Stimulating Hormone Levels with Microvascular Complications in Type 2 Diabetes Patients. Medical science monitor: international medical journal of experimental and clinical research.

[17] Petrosyan L (2015). Relationship between high normal TSH levels and metabolic syndrome components in type 2 diabetic subjects with euthyroidism. Journal of clinical & translational endocrinology.

[18] Zhang Y, Lu P, Zhang L, Xiao X (2015). Association between lipids profile and thyroid parameters in euthyroid diabetic subjects: a cross-sectional study. BMC endocrine disorders.

[19] Kliger AS (2013). KDOQI US commentary on the 2012 KDIGO Clinical Practice Guideline for Anemia in CKD. American journal of kidney diseases: the official journal of the National Kidney Foundation.

[20] Inker LA (2014). KDOQI US commentary on the 2012 KDIGO clinical practice guideline for the evaluation and management of CKD. American journal of kidney diseases: the official journal of the National Kidney Foundation.

[21] Garber JR (2012). Clinical practice guidelines for hypothyroidism in adults: cosponsored by the American Association of Clinical Endocrinologists and the American Thyroid Association. Thyroid: official journal of the American Thyroid Association.

[22] National Kidney, F. (2012). KDOQI Clinical Practice Guideline for Diabetes and CKD: 2012 Update. American journal of kidney diseases: the official journal of the National Kidney Foundation.

[23] Gu Y (2017). The Relationship Between Thyroid Function and the Prevalence of Type 2 Diabetes Mellitus in Euthyroid Subjects. The Journal of clinical endocrinology and metabolism.

[24] Altinova AE (2006). Adiponectin levels and cardiovascular risk factors in hypothyroidism and hyperthyroidism. Clinical endocrinology.

[25] Knudsen N (2005). Small differences in thyroid function may be important for body mass index and the occurrence of obesity in the population. The Journal of clinical endocrinology and metabolism.

[26] Surks MI (2004). Subclinical thyroid disease: scientific review and guidelines for diagnosis and management. Jama.

[27] Nishimura T, Tanaka M, Sekioka R, Itoh H (2015). Serum bilirubin concentration is associated with eGFR and urinary albumin excretion in patients with type 1 diabetes mellitus. Journal of diabetes and its complications.

[28] Lupoli R (2014). Effects of treatment with metformin on TSH levels: a meta-analysis of literature studies. The Journal of clinical endocrinology and metabolism.

[29] Cappelli C (2012). Thyreotropin levels in diabetic patients on metformin treatment. European journal of endocrinology.

[30] Feely J, Isles TE (1979). Screening for thyroid dysfunction in diabetics. British medical journal.

[31] Iglesias P, Diez JJ (2009). Thyroid dysfunction and kidney disease. European journal of endocrinology.

[32] Rai S (2013). Thyroid function in type 2 diabetes mellitus and in diabetic nephropathy. Journal of clinical and diagnostic research: JCDR.

[33] Wu J, Li X, Tao Y, Wang Y, Peng Y (2015). Free Triiodothyronine Levels Are Associated with Diabetic Nephropathy in Euthyroid Patients with Type 2Diabetes. International journal of endocrinology.

[34] Boucai L, Hollowell JG, Surks MI (2011). An approach for development of age-, gender-, and ethnicity-specific thyrotropin reference limits. Thyroid: official journal of the American Thyroid Association.

[35] Napoli R (2001). Impact of hyperthyroidism and its correction on vascular reactivity in humans. Circulation.

[36] Isumi Y (1998). Regulation of adrenomedullin production in rat endothelial cells. Endocrinology.

[37] Ojamaa K, Klemperer JD, Klein I (1996). Acute effects of thyroid hormone on vascular smooth muscle. Thyroid: official journal of the American Thyroid Association.

[38] Yilmaz MI (2011). Low triiodothyronine alters flow-mediated vasodilatation in advanced nondiabetic kidney disease. American journal of nephrology.

[39] Volzke H (2009). Are serum thyrotropin levels within the reference range associated with endothelial function?. European heart journal.

[40] Siddiqi FS, Advani A (2013). Endothelial-podocyte crosstalk: the missing link between endothelial dysfunction and albuminuria in diabetes. Diabetes.

[41] Lin Y, Sun Z (2011). Thyroid hormone ameliorates diabetic nephropathy in a mouse model of type II diabetes. The Journal of endocrinology.

[42] Shang G (2013). 3,5-Diiodo-l-thyronine ameliorates diabetic nephropathy in streptozotocin-induced diabetic rats. Biochimica et biophysica acta.

[43] Xiang GD (2006). Impairment of endothelium-dependent arterial dilation in Hashimoto’s thyroiditis patients with euthyroidism. Clinical endocrinology.

[44] American Diabetes, A. (2016). 2. Classification and Diagnosis of Diabetes. Diabetes care.

[45] Sobel R (1990). Screening for thyroid disease. Israel journal of medical sciences.

[46] Moura Neto A (2016). Relation of thyroid hormone abnormalities with subclinical inflammatory activity in patients with type 1 and type 2 diabetes mellitus. Endocrine.

